# Screening for Colorectal Cancer Leading into a New Decade: The “Roaring ‘20s” for Epigenetic Biomarkers?

**DOI:** 10.3390/curroncol28060411

**Published:** 2021-11-20

**Authors:** Hélder Almeida-Lousada, André Mestre, Sara Ramalhete, Aryeh J. Price, Ramon Andrade de Mello, Ana D. Marreiros, Ricardo Pires das Neves, Pedro Castelo-Branco

**Affiliations:** 1 Faculty of Medicine and Biomedical Sciences (FMCB), Campus de Gambelas, University of Algarve, 8005-139 Faro, Portugal; helderlousada@sapo.pt (H.A.-L.); andre_mestre94@hotmail.com (A.M.); saravramalhete@gmail.com (S.R.); ramondemello@gmail.com (R.A.d.M.); ammarreiros@ualg.pt (A.D.M.); 2 Algarve Biomedical Center Research Institute (ABC-RI), 8005-139 Faro, Portugal; 3 School of Law, University of California, Berkeley, CA 94704, USA; price.aryeh@berkeley.edu; 4 Division of Medical Oncology, Escola Paulista de Medicina, Federal University of São Paulo (UNIFESP), São Paulo 04037-004, Brazil; 5 Precision Oncology & Health Economics Group (ONCOPRECH), Post-Graduation Program in Medicine, Nine of July University (UNINOVE), São Paulo 01525-000, Brazil; 6 CNC—Center for Neuroscience and Cell Biology, CIBB—Centre for Innovative Biomedicine and Biotechnology, University of Coimbra, 3004-517 Coimbra, Portugal; 7 IIIUC—Institute of Interdisciplinary Research, University of Coimbra, 3004-517 Coimbra, Portugal; 8 Champalimaud Research Program, Champalimaud Center for the Unknown, 1400-038 Lisbon, Portugal

**Keywords:** colorectal cancer, epigenetic testing, screening, diagnosis, CRC biomarkers

## Abstract

Colorectal cancer (CRC) has an important bearing (top five) on cancer incidence and mortality in the world. The etiology of sporadic CRC is related to the accumulation of genetic and epigenetic alterations that result in the appearance of cancer hallmarks such as abnormal proliferation, evasion of immune destruction, resistance to apoptosis, replicative immortality, and others, contributing to cancer promotion, invasion, and metastasis. It is estimated that, each year, at least four million people are diagnosed with CRC in the world. Depending on CRC staging at diagnosis, many of these patients die, as CRC is in the top four causes of cancer death in the world. New and improved screening tests for CRC are needed to detect the disease at an early stage and adopt patient management strategies to decrease the death toll. The three pillars of CRC screening are endoscopy, radiological imaging, and molecular assays. Endoscopic procedures comprise traditional colonoscopy, and more recently, capsule-based endoscopy. The main imaging modality remains Computed Tomography (CT) of the colon. Molecular approaches continue to grow in the diversity of biomarkers and the sophistication of the technologies deployed to detect them. What started with simple fecal occult blood tests has expanded to an armamentarium, including mutation detection and identification of aberrant epigenetic signatures known to be oncogenic. Biomarker-based screening methods have critical advantages and are likely to eclipse the classical modalities of imaging and endoscopy in the future. For example, imaging methods are costly and require highly specialized medical personnel. In the case of endoscopy, their invasiveness limits compliance from large swaths of the population, especially those with average CRC risk. Beyond mere discomfort and fear, there are legitimate iatrogenic concerns associated with endoscopy. The risks of perforation and infection make endoscopy best suited for a confirmatory role in cases where there are positive results from other diagnostic tests. Biomarker-based screening methods are largely non-invasive and are growing in scope. Epigenetic biomarkers, in particular, can be detected in feces and blood, are less invasive to the average-risk patient, detect early-stage CRC, and have a demonstrably superior patient follow-up. Given the heterogeneity of CRC as it evolves, optimal screening may require a battery of blood and stool tests, where each can leverage different pathways perturbed during carcinogenesis. What follows is a comprehensive, systematic review of the literature pertaining to the screening and diagnostic protocols used in CRC. Relevant articles were retrieved from the PubMed database using keywords including: “Screening”, “Diagnosis”, and “Biomarkers for CRC”. American and European clinical trials in progress were included as well.

## 1. Introduction

Colorectal cancer (CRC) is the third most prevalent cancer in males and females and the second and fourth (males and females) principal cause of cancer death in the world, accounting for about 1,755,700 new cases and 1,402,750 deaths in 2018 alone. It is a problem not only in poor undeveloped countries (60% deaths) but it has also been associated with infection by pathogens (e.g., papilloma virus) in countries undergoing rapid societal and economic changes and in high-income countries [1].

CRC cases and deaths could be avoided through improvements in screening methodology [2]. This would benefit from identifying and testing key biomarkers of the molecular, epigenetic, and genetic CRC pathways in order to establish the main marks of CRC onset and evolution.

### 1.1. Genetic and Molecular Pathogenesis of CRC

CRC develops over a long period of time, where the accumulation of epigenetic and genetic errors leads to histological changes that result in macroscopic lesions with the characteristics of CRC, namely, uncontrolled cell proliferation and tissue invasiveness by continuity or at a distance. The origin of these alterations includes lifestyle exposure to mutagens and heritable factors. Mutations in three important gene clusters, APC/beta-Catenin, k-Ras and TP53, are sufficient to initiate CRC [3,4,5].

CRC genesis proceeds from an early nascent phase through a period of rapid molecular diversification, culminating in extensive invasion and metastasis. Most of the culpable driver genes integrate a limited number of key signal transduction pathways, which must be disabled in order to sustain neoplastic clonal expansion. In CRC, recurring pathways include wnt/wingless (via APC and CTNNB1/β catenin), EGF (via KRAS, BRAF, et cetera), and TGF β (via SMAD4, p53) [3,6,7].

CRC manifests in sporadic and hereditary forms. Although CRC family history can be detected in about 25% of cases, most arise sporadically. Three different inciting events have been related to the development and progression of CRC: chromosomal instability (CIN), microsatellite instability (MSI), and the CpG island methylator phenotype (CIMP).

CIN tumors are the most frequent (70%) and represent the sum of numerical (aneuploidies) or structural chromosomal abnormalities, resulting in karyotypic variations in colonic mucosa. It is characterized by recurrent loss-of-heterozygosity at tumor suppressor loci and chromosomal reorganizations [7]. These genetic changes originate in the deregulation of many genes with important roles in the control of DNA homeostasis, such as tumor suppressor genes (TSGs) and the well-known oncogenes APC, KRAS, and TP53 [5,8].

CRC can occur in inherited tumors, such as familial adenomatous polyposis, where hundreds to thousands of adenomatous colorectal polyps may develop, usually in the second decade of life [9]. On the other hand, CIN is also linked to most sporadic aneuploid CRCs [4].

DNA replication errors are another important mechanism leading to genomic instability. An example of this is the MSI pathway that originates in the deregulation of the DNA Mismatch Repair (MMR). DNA replication errors are corrected by two mechanisms: the DNA polymerase has its own intrinsic revision process, and then the MMR corrects mismatches between bases and insertion/deletion loops, avoiding permanent mutations. In this protection mechanism, the first step is the recognition and binding of the MSH2/MSH6 complex or the MSH2/MSH3 complex to DNA errors during replication. In the second step, Mutα recruits the MLH1/PMS2 complex that promotes error repair. MSI occurs when one of these proteins fails, leading to indel-like mutations (slippage of the microsatellite DNA strand during replication). When these indels are located inside DNA coding regions, they can lead to further mutations, which are the origin for the downstream production of abnormal protein products [8].

MSI tumors tend to appear in the right side of the colon, generally have high histological grades, a mucinous phenotype, and are often diagnosed at earlier TNM classification stages when compared to CIN-induced cancers [10]. The TNM system is an international histopathological classification to characterize a cancer, where T represents the size of the primary cancer (in CRC, this is the depth of the invasion, regardless of the overall tumor size), N represents the invasion of regional lymph nodes by the primary cancer, and M represents the invasion of organs other than the organ where the primary cancer developed, known as metastasis. This system allows the definition of therapeutical protocols and helps in the determination of the patient’s prognosis and survival [11].

CIMP tumors arise from the widespread hypermethylation of CpG islands and up to 40% of them are located in the proximal segment of the colon. They can be divided into CIMP-high harboring BRAF mutations and MLH1 methylation, or CIMP-low, with *KRAS* mutations [12,13]. The poor prognosis in CIMP-high tumors is putatively attributable to BRAF mutations.

In general, the three pathways are rarely found to co-exist. Nonetheless, a complex interface has been documented in some rare tumors, including CIMP and MSI, as a result of MLH1 promoter hypermethylation [14,15].

**Figure 1:**
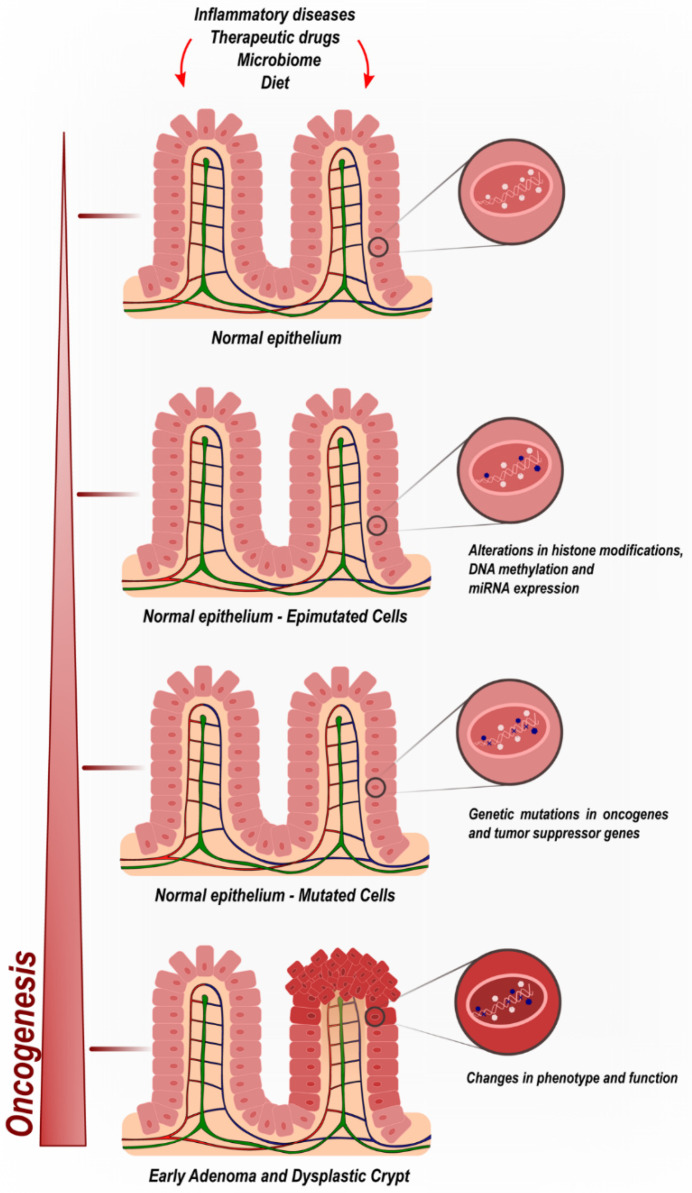
Colorectal cancer oncogenic progression by cumulative epigenetic and genetic deregulatory mechanisms.

The gradual, stepwise, and cumulative process of CRC tumorigenesis is the result of not only genetic but also epigenetic modifications (Figure 1). Such molecular alterations transform the normal colonic mucosa into CRC. Epigenetics encompasses mechanisms capable of modifying gene expression levels without disrupting the underlying DNA sequence. There is extensive diversity in the range of epimutations identified to date, including but not limited to DNA methylation, histone modifications, and noncoding RNA regulation [16,17].

### 1.2. Epigenetic Mechanisms in CRC—DNA Methylation

DNA methylation usually occurs in CpG islands. These are regions of the genome that contain a large number of CpG dinucleotide repeats. In mammalian genomes, CpG islands usually extend for 300–3000 base pairs. They are located within and close to sites of about 40% of mammalian gene promoters and typify one of the most extensively studied epigenetic marks [18]. The addition of a methyl group (CH3) to the 5′ position of the cytosine pyrimidine ring, creating 5-methylcytosine (5-mC), is known as methylation. The three-dimensional structure of chromatin can be affected by the methylation of CpG islands in a gene’s promoter, increasing or decreasing the degree of DNA folding. In this way, the three-dimensional alterations of the DNA chain can increase or decrease the expression of one or more genes.

Promoter methylation has traditionally been associated with gene silencing and promoter demethylation with upregulated gene expression, though this dogma has been challenged by compelling counterfactuals in recent years [19,20]. In CRC, both DNA hypermethylation and hypomethylation events have been documented [21]. Examples of targets for methylation-mediated dysregulation include E-cadherin and P-cadherin, which are normally expressed in the basal layer of squamous epithelia and not found in the normal colonic mucosa. They are expressed in all mucous membranes subject to biological stress. P-cadherin has been expressed since the beginning of the precursory changes in cancer and neoplasms. Demethylation of the P-cadherin promoter has been found to be a frequent trait in the progression of adenomatous lesions to colorectal carcinoma [22].

### 1.3. Epigenetic Mechanisms in CRC—MicroRNA Regulation

MicroRNAs (miRNAs) are single-stranded small RNAs comprising 18–25 nucleotides that prevent the translation of specific messenger RNAs, either by direct interference or by promoting target mRNA degradation [23]. There are ~1900 miRNA-encoding genes present in the human genome; of these, ~250 miRNAs show alterations in expression and/or function in CRC [24].

One study showed that, in CRC, about 33% of miRNAs were under-expressed and the rest were over-expressed, which may indicate that the role of miRNAs in CRC may be more oncogenic than suppressive. Moreover, this seems to indicate that the production of these molecules is enhanced in CRC [25,26].

Multiple studies have demonstrated that miR-143 and miR-145 function as tumor suppressors in CRC. A highly relevant microRNA in CRC is the oncogenic microRNA, miR-21, which is elevated in CRC. Other microRNAs altered in CRC include the miR-17Y92 cluster, miR-106a, miR-31, miR-181b, miR-183, miR-135a/b, the miR-200a/b/c family, miR-203, and miR-224 [23,27]. 

### 1.4. Epigenetic Mechanisms in CRC—Histone Modifications

Histone post-translational modifications (PTMs) include acetylation, methylation, ADP ribosylation, SUMOylation, biotinylation, crotonylation, citrulation, ubiquitination, phosphorylation, and isomerization [27,28]. These can happen in different amino acid residues at several positions, although they are most common at histone N-terminal tails [29,30]. Mounting evidence suggests that aberrant histone lysine acetylation is a critical epigenetic driver of malignant transformation [31,32]. Histone lysine acetylation is regulated mostly by Histone acetyltransferases (HATs) and Histone deacetylases (HDACs). Cancers are, in most cases, characterized by marked histone deacetylation [31,32,33]. Particularly, HDAC2 upregulation is related to early events in CRC, while HDAC1, 3, 5, and 7 have been found to be upregulated in advanced CRC. The analyses of chromatin modifications in Transmission Electron Microscopy images of nuclei from CRC samples has shown that HDAC2 significantly increased in the adjacent mucosa of patients with adenomatous polyps (precancerous lesions) when compared to the control group of patients, suggesting that this histone chromatin modification is present in early stages of CRC [34,35].

## 2. Objectives

The aim of this study is to catalogue the techniques and tests comprising the status quo for the screening and diagnosis of CRC as we progress through the 2020s. We describe and make the case for novel genetic and epigenetic tools that promise noninvasive CRC screening with earlier and more effective readouts.

## 3. Materials and Methods

This work is the result of a comprehensive literature review. We searched for ongoing clinical trials in the United States (accessed on 12 May 2021, https://clinicaltrials.gov/) and Europe (accessed on 12 May 2021, https://www.clinicaltrialsregister.eu/). Relevant articles on the subject were retrieved from PubMed databases using the keywords:

“Colorectal cancer”; “Epigenetic and biomarkers and colorectal cancer”; “Diagnostic and colorectal cancer”; “Screening and Colorectal cancer”; “Epigenetic and colorectal cancer”; “Epigenetic and screening and colorectal cancer”; “Colorectal cancer and histology”.

To minimize gaps in data capture, the reference sections of the articles identified in our primary searches were mined for potentially overlooked citations. 

## 4. Techniques Used for Screening and Diagnosis of CRC

CRC high-risk individuals with CRC family histories undergo colonoscopy and diagnostic histopathology. Appropriate CRC screening may be more valuable for detection of early-stage tumors and precancerous lesions in asymptomatic individuals with an average risk of developing the disease [36].

**Figure 2:**
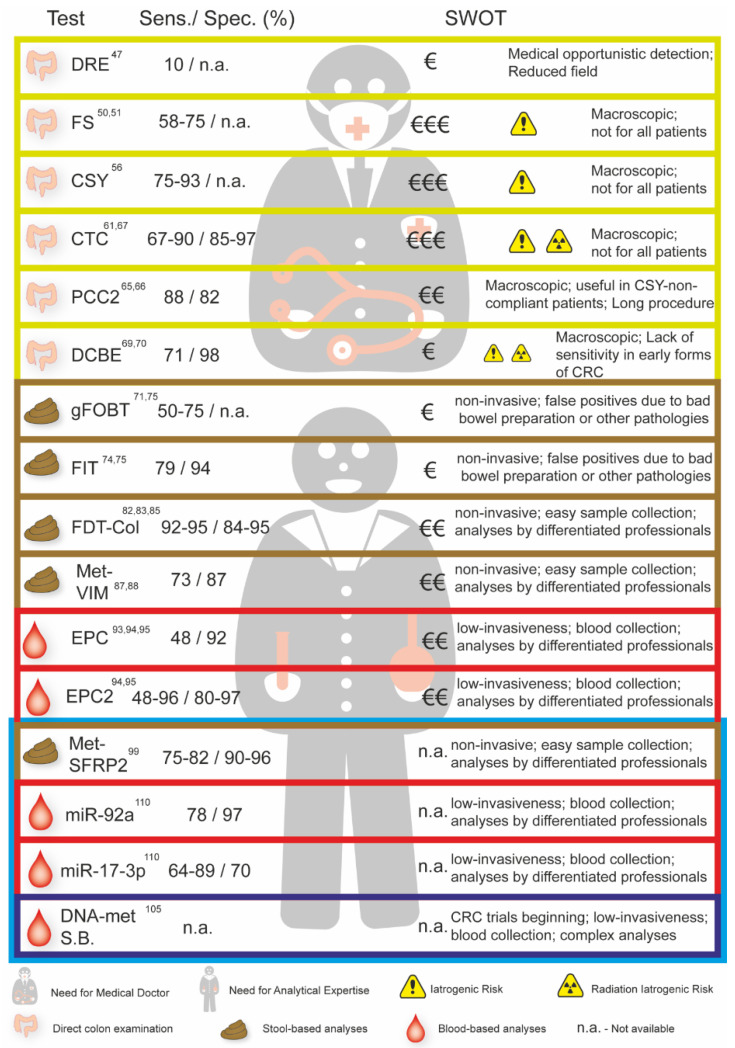
Sensitivity, specificity, and analyses of the Strengths, Weaknesses, Opportunities, and Threats (SWOT) of the different CRC screening tests currently available and in clinical trials (blue box). SWOT analyses including the costs, strengths, and weaknesses of each screening test are summarized. DRE—Digital Rectal Examination; FS—Flexible Sigmoidoscopy; CSY—Colonoscopy; CTC—Colonography; PCC2—Colon Capsule endoscopy; DCBE—Double Contrast Barium Enema; FOBT—Fecal Occult Blood; FIT—Fecal Occult Blood Immunoassay; FDT-Col—Fecal DNA Testing; Met-VIM—Methylated VIM; EPC—Septin 9 Assay: EPC 2—Septin 9 Assay (second generation); Met SFRP2—Methylated SFRP2; miR-92a—Methylation miR-92a; miR-17-3p—Methylation miR-17-3p; DNA-met S.B.—Blood Based DNA Methylation Beginning Trials.

CRC prevalence and its insidious natural history make it a prime candidate for screening when compared to other types of cancers. Indeed, CRC can take over two decades to progress from an early sub-clinical neoplasia to an advanced disease and symptomatic presentation. However, evidence on screening’s effectiveness is incomplete, and the cost–benefit ratio has yet to be fully elucidated [36] (Figure 2).

New milestones in extending patient survival may be attributable to the increased use of innovative imaging techniques, such as positron emission tomography, which can improve the precision of staging and inform treatment decisions [37].

Numerous factors modify an individual’s probability of developing CRC relative to the estimated 6% in the average-risk population [38]. These factors include inflammatory bowel disease (chronic ulcerative colitis and Crohn’s disease), familial polyposis syndromes (for example, inherited adenomatosis of the rectum and colon), the Gardner, Turcot, and Oldfield familial cancer syndromes, a history of CRC in a first-degree relative, and a previous personal history of cancer [38,39].

The primary risk is found in patients with familial adenomatosis, wherein they have a high probability of developing CRC at early age. Overall, heritable forms of polyposis and nonpolyposis syndromes are responsible for approximately 5% of all colonic cancers [40,41].

Furthermore, an individual with a history of at least 10 years of ulcerative colitis involving more colonic mucosa than rectal, possesses a 20-fold higher risk for developing CRC [36]. Moreover, the risk for individuals with ulcerative colitis involving only the rectal mucosa is also higher than that of the average population [38].

The heightened risk for CRC in patients with Crohn’s disease is less than in patients with ulcerative colitis, but greater still than the average-risk population, and it rises dynamically over time.

Relevant family history, excluding familial polyposis (such as having a first-degree relative with CRC, hereditary nonpolyposis colorectal cancer syndrome, and MutY homolog polyposis), increases the risk of CRC incidence in 1.8 at 3.8. The existence of a first-degree relative with CRC confers a two-to-three-fold higher risk when compared to the average [42].

Screening asymptomatic individuals who are at an average or high risk improves cancer detection at early stages, thereby decreasing mortality. However, fewer than 30% of these individuals have requested a screening test for CRC. This low rate has been ascribed to resistance on the part of physicians, patients, and health care organizations.

There are multiple screening tests with distinct sensitivity and specificity profiles, each of them with advantages, disadvantages, and iatrogenic risks. Hence, the process of informed decision making on which test to use is complex and requires weighing diagnostic value, costs, patient preference, and technical aspects such as availability of the technique or specialized medical personnel [43].

Overall, the sooner the screening test is performed, the more effective it will be in patient survival. However, screening tests specifically designed for each risk category are still lacking.

Most studies show convincing evidence that screening for CRC in adults aged 50 to 75 years old reduces mortality [44].

There are no head-to-head studies demonstrating that any existing screening methods have superior efficacy over others, although existing tests have varying degrees of evidence supporting their utility, as well as different strengths and limitations [44,45,46].

### 4.1. Digital Rectal Examination

A digital rectal examination (DRE) may be done as part of a physical examination to detect rectal cancers. However, the sensitivity of DRE is low, as fewer than 10% of CRCs are within reach of the examining finger [47]. Not only is DRE considered to be a poor indicator of rectal cancer, but its ability to detect colon cancer is virtually non-existent [48].

### 4.2. Sigmoidoscopy

A flexible sigmoidoscopy every 3 to 5 years is recommended for men and women with average risk between 50 and 75 years of age. It consists of a camera on a flexible tube introduced through the anus to examine the rectum and, at most, the first third of the large colon. Any polyps detected are recorded, biopsied, and usually followed by a prompt referral for colonoscopy [44].

Multiple clinical trials reported an adjusted odds ratio of 0.41 (CI, 0.25 to 0.69), suggesting that sigmoidoscopy screening reduced the risk of death by 59% [49].

Numerous randomized clinical trials evaluating one-time or two-time flexible sigmoidoscopy (*n* = 458,002) were associated with reduced CRC-specific mortality, compared to no screening (incidence rate ratio, 0.73; 95% CI, 0.66–0.82). The sensitivity of flexible sigmoidoscopy for screening CRC was 58% to 75%, based on small numbers of colorectal lesions, with an estimated sensitivity of 72% to 86% for advanced CRC [50,51,52].

### 4.3. Colonoscopy

Colonoscopy is the most sensitive and specific test available for screening for CRC and adenomas, but it is operator-dependent and poses a higher iatrogenic risk. The risks for the patient associated with colonoscopy are essentially bleeding and intestinal perforation, mainly, but not only, when performing instrumented colonoscopy for polyp excision or mucosal biopsy [37,51,53].

There is currently some debate to determine whether colonoscopy is the best method for screening for CRC, or whether it should only be used for diagnosis after screening with non-invasive tests. In addition to the iatrogenic risks inherent to the procedure, it also requires highly trained physicians, and suffers from variable compliance of the patients, often requiring anesthesia or deep sedation and being dependent on the adherence of the patient to the procedure and proper bowel preparation [36,54].

Adverse events from colonoscopies in asymptomatic individuals included perforations at an estimated risk of four per 10,000 (95% confidence interval) and major hemorrhage of eight per 10,000 (95% confidence interval) with screening colonoscopy [55].

Moreover, the sensitivity of colonoscopy to detect adenomas of 6 mm or larger ranged from 75% (95% Colonoscopy, 63–84%) to 93% (95% Colonoscopy, 88–96%) [56].

The effectiveness and, in part, the safety of colonoscopy depends on the doctor’s training, the patient’s engagement and education, and also the quality of colon preparation. To optimize results, patients must be part of the process and receive clear, simple information and instructions in their native language [49]. Additionally, colonoscopy frequently requires sedation and involves skilled support personnel. It is more expensive and has a higher risk for procedural complications than other screening tests, particularly when polypectomy is performed. Death, infections, sedation-related events, and chemical colitis are less frequent but important to consider. Bleeding requiring hospitalization, strokes, myocardial infarctions, and thrombophlebitis have also been reported [36,57].

As a diagnostic tool, perforation and major bleeding rates are low. However, using colonoscopy as a therapeutic procedure presents higher complication rates. Deaths rarely occur, though they have a higher incidence in studies with older and more symptomatic patient cohorts [58].

Nevertheless, despite the potential complications and relative high costs, colonoscopy presents superior sensitivity and specificity.

### 4.4. CT Colonography

CT colonography (CTC), or virtual colonoscopy, is a radiographic exam where multiple radiological acquisitions are made by planes, which are then treated by specific software resulting in the presentation of bi- or three-dimensional images of the abdomen on a monitor, allowing for its evaluation by a physician gastroenterologist to identify lesions of the colon mucosa. It is advisable in instances of incomplete colonoscopy, patient refusal, or the presence of additional risk factors such as recent colectomy or bowel stenosis [59].

The execution of CTC involves a double contrast that is obtained by bowel preparation based on laxatives and diet, after which a radiopaque contrast medium is ingested. Colonic insufflation functions as a radio-transparent contrast and allows the creation of a virtual space in the colon’s lumen. Insufflation of the colon with CO2 poses a minor risk of intestinal rupture. After these procedures, radiographic image capture begins. These images are generally acquired in two positions, supine and prone, to obtain good images of the entire mucosa. Only in very large patients will it be necessary to also use lateral decubitus positions. After the exam, the images are processed by software that generates bi- and three-dimensional images that allow the assessment of lesions larger than one centimeter and smaller than one centimeter, respectively [43,50,58].

CTC is a marginally invasive imaging technique with coverage of the entire colon and rectum that shows high individual sensitivity. Performing CTC without previous bowel preparation decreases sensitivity and specificity for lesions larger than 1 cm to 67–90% when compared with 85–97% obtained with preparation. 

One of the problems related to CTC for CRC screening is extra colonic findings, which are frequent, occurring in 40–70% of exams. There is data showing that 5–37% of these findings are subsequently followed up and only 3% need therapy. These numbers show a unique risk of this screening method for CRC, which is the over-diagnosis and over-treatment associated with these findings, which are often innocuous but generate anxiety in the patient and can result in iatrogenicity [44,60].

New technological developments have rendered the risk of ionizing radiation from CTC extremely low and likely insignificant [61].

CTC screening could be particularly appropriate for patients between 50 and 65-years old who seek a noninvasive test with very high sensitivity. Available evidence suggests that asymptomatic patients with a positive fecal immunochemical DNA test and a negative high-quality CTC do not need colonoscopy nor evaluation of the remainder of the gastrointestinal tract.

CTC shows better sensitivity and specificity values in the detection of CRC. Moreover, patients tolerate CTC better. With new low-radiation methods, CTC has practically replaced the barium enema in CRC screening [62]. Advantages of CTC include a lower risk of perforation compared to colonoscopy and CTC has a sensitivity of at least 90% for adenomas ≥1 cm in size [56,61,62]. When comparing CTC with colonoscopy, we found that colonoscopy maintains an advantage in detecting infra-centimeter lesions and flat and serrated lesions [63]. The detection of mostly clinically non-relevant extra-colon findings and radiation exposure together with the lower capacity to detect the lesions mentioned above are the main disadvantages of CTC [64].

### 4.5. Colon Capsule Endoscopy

Colon Capsule Endoscopy (CCE) (Pillcam COLON, Dado Imaging Ltd., Yoqneam, Israel) is an imaging test in which the bowel lumen is imaged by a device with two digital cameras that is ingested by the patient. This device captures images at a rate of 4 to 35 images per second, depending on the speed of the device, for a period that can reach 10 h of recording. The device is expelled, and images are compiled and viewed on a monitor by an experienced gastroenterologist to identify mucosal lesions [65]. In addition to polyps or masses, CCE was shown to detect other illnesses, including diverticulosis and erythema/inflammation, that were not diagnosed by colonoscopy in a randomized study [66].

The use of CCE is advised in cases of colonoscopy contraindication, colonoscopy failure, or in patients reluctant to perform colonoscopy. In the United States, the Food and Drug Administration has approved Pillcam COLON 2 (second generation) for patients who have had an incomplete colonoscopy or with other risk conditions for colonoscopies [63,65]. A recent prospective study conducted by Doug Rex on 884 patients, comparing the accuracy of PillCam COLON 2 to that of optical colonoscopy, demonstrated 88% sensitivity and 82% specificity in detecting adenoma ≥ 6 mm in an average-risk screening population [65].

An advantage of capsule endoscopy, compared to other minimally invasive methods, is the absence of radiation exposure and that it is well-tolerated by most patients. Disadvantages of capsule endoscopy include the need for complex bowel preparation and the risk of capsule retention, which constitutes a very rare event but may require surgical or endoscopic removal of the device [67].

Adverse outcomes of varying seriousness (mild to moderate), have been reported, but in most of these cases the adverse outcome was resolved spontaneously in 48 h [68]. Another multicenter study showed a sensitivity as high as 84% and 88% for 6-mm and 10-mm polyps, respectively, when comparing second-generation CCE to colonoscopy [66].

### 4.6. Double Contrast Barium Enema 

Double Contrast Barium Enema (DCBE) is a minimally invasive imaging screening method for CRC, where Barium and air are used to profile the rectum and colon on an X-ray. DCBE showed a sensitivity and specificity of 71% and 98% for the detection of colonic lesions larger than 7 mm [69]. These results are congruent with a meta-analysis comparing the results of DCBE to that of CTC, showing CTC to be more sensitive and specific than DCBE for large polyps [70].

DCBE has good sensitivity and specificity for detecting CRC at an advanced stage but detects infra-centimeter-sized precursor lesions with lower sensitivity values than other methods, such as colonoscopy or CTC. In addition, DCBE involves a subsequent colonoscopy if lesions are detected for biopsy. For these reasons DCBE is falling out of favor in CRC detection [62,69,70].

The described techniques used for CRC screening and diagnosis have their drawbacks regarding costs, sensitivity, specificity, and low rates of compliance from the average-risk population. Most of them can detect lesions bigger than 7 mm and they should be chosen depending on patient eligibility. From all the techniques discussed above, colonoscopy is the method that has the best sensitivity for smaller lesions. The detection of this type of lesion is more challenging for imaging techniques and can benefit from molecular biomarkers, which can be instrumental in early CRC detection. They also have lower costs, decreased invasiveness, and lack the requirement for specialized clinicians.

## 5. Biomarkers Used for Screening and Diagnosis of CRC

Biochemical biomarkers, including fecal occult-blood tests (guaiac and fecal immunochemical tests) have favorable sensitivity and specificity for screening CRC in initial TNM stages. However, they can only detect the histological manifestation upon the presence of polyps or adenomas that bleed into the colon and are ineffective for CRC lesions without bleeding. To overcome this hurdle, epigenetic-based biomarkers have been under development as they can distinguish other causes of bleeding unrelated to CRC that include antiplatelet or anticoagulant medication, peptic ulcers, or bowel angiomas.

### 5.1. Fecal Screening Tests

The ideal method for CRC screening would be cheap and non-invasive, with good acceptance by patients, and with high sensitivity and specificity values. The development of tests performed on small stool samples that detect indirect signs of CRC, such as the presence of occult blood or genetic and epigenetic alterations, that are related to the presence of CRC, has been done with good results [67].

#### 5.1.1. Fecal Occult-Blood Tests—gFOBT and FIT

There are two tests available that assess the presence of occult blood in feces as an indirect marker of CRC. The guaiacol fecal occult-blood test (gFOBT) is based on the detection of the heme group through its peroxidase activity. The guaiac test is not specific for human blood and has a sensitivity of 33–50% for CRC screening. Recently, a test based on the same detection method (Hemoccult SENSA, Beckman Coulter) with an improved sensitivity for CRC screening (sensitivity 50–75%) has come into the market.

The fecal immunochemical test (FIT) uses antibodies that are specific for human hemoglobin, albumin, and other blood components such as globulin, and is more sensitive than the gFOBT test [71,72,73].

FIT testing is simpler and easier to perform, as it has no dietary or medication restrictions. Additionally, its overall precision for CRC detection is 95% with 79% sensitivity and 94% specificity, as previously shown [73,74]. Furthermore, it has greater sensitivity in detecting advanced adenomas and CRC when compared to gFOBT [75].

It has been shown that patients with positive fecal occult-blood tests have a higher risk of dying from CRC (Risk 7.79: 95% CI, 6.13 to 9.89), but also from other diseases, such as cardiovascular, respiratory, blood, or other gastrointestinal diseases other than CRC [76].

Colonoscopy or other visual exams, such as CTC or CCE, adjusted to account for clinical condition and patient preferences, should be recommended to individuals with positive tests. 

Most advanced adenomas (≥10 mm in diameter or with villous histologic features or high-grade dysplasia) are undetectable or poorly detected by examinations of fecal occult-blood tests. [69,73,76].

The gFOBT test based on the peroxidation reaction shows an increase in false positives if the patient ingests animal products (mainly undercooked red meat) and some types of vegetables before and during the test. On the other hand, the intake of vitamin C and other antioxidants promotes the increase of false negatives in this type of test [77,78,79].

#### 5.1.2. Fecal DNA Testing

In 2014, the FDA authorized the introduction of the Cologuard^®^ test, a DNA test done in stool that targets multiple genes that are frequently altered in colon shedding cells [80]. This multi-target test is non-invasive, easy to perform without iatrogenic issues or dietary restrictions, and only requires a stool sample [81].

Stool DNA tests are supported by research findings that well-defined mutations are associated with CRC and cellular DNA is expelled in stool at levels detectable by polymerase chain reaction (PCR) [82,83]. It is a composite test that includes an immunochemical analysis, methylated biomarkers (ex: BMP3 and NDRG4), and genetic mutations (e.g., KRAS and ACTB) associated with CRC [80,81,84].

A multicenter study involving nearly 10,000 patients showed that the multi-target fecal DNA test had a higher sensitivity than FIT for screening advanced adenomas and CRC. However, this higher sensitivity of Cologuard^®^ is associated with a lower specificity. The sensitivity for CRC of the fecal DNA test was 92% (95% CI, 84–97%) and 42% (95%, CI 39–46%) for advanced adenoma. This study used colonoscopy as the gold standard [48,82].

Regrettably, fecal DNA testing detected fewer than half of all large, advanced adenomas (42.4%), and 23.8% with FIT limiting its utility to a preventive role [85].

A recent study compared the cost-effectiveness of Cologuard^®^ CRC screening versus FIT and colonoscopy. After normalization, both FIT and colonoscopy were shown to be cheaper and more effective for detecting CRC [86].

#### 5.1.3. Methylated Vimentin

Another fecal DNA marker is the Vimentin (VIM) gene. Methylated VIM occurs in CRC and is detected in fecal DNA with good sensitivity. It was shown that Vimentin promoter hypermethylation in patients with CRC has a specificity of 100% and a sensitivity of 60%; showing that this epigenetic marker has a positive predictive value of 100% [87]. Another study showed that testing based on altered methylation of the vimentin promoter alone had a sensitivity of 72.5% and specificity of 86.9% [88].

Several studies have shown the relationship between hypermethylation of the vimentin promoter in desquamated cells present in feces and CRC, suggesting its usefulness for screening CRC in patients. These and other studies culminated in the development of ColoSure™ (Lab Corp, Burlington, NC, USA), one of the first tests based on epigenetic markers for screening for CRC [89].

Weaknesses of fecal DNA testing include its expensive cost, the inconvenience of stool sampling and delivery to the lab, and the need for a colonoscopy if the test is positive [67].

### 5.2. Blood Screening Tests

Biomarkers for CRC in blood or blood serum are at the beginning of their development; however, they may prove valuable for CRC screening due to the simplicity of sampling, low costs, and easy adherence by patients.

Septins are a group of proteins involved in maintaining structural integrity during cell division. Septins are organized into heteromers of six to eight subunits. Two molecules of the SEPT2, SEPT6, SEPT7, and SEPT9 subunits each make up the octamer [90]. SEPT9 occupies a terminal position in the complex and stabilizes the octamer. This subunit plays an important role in cell separation at the end of mitosis [85,86]. Cytokinesis may be extremely affected if abnormal SEPT9 or no SEPT9 is expressed. As a result of this function, SEPT9 promoter hypermethylation and the resultant transcriptional compromise may be a key driver of CRC carcinogenesis [91,92].

Hypermethylated Septin9 fragments can be found in tumor DNA that passes into the bloodstream from all colonic anatomical segments. It is the only gene that is used in commercially available plasma kits (Epi ProColon; Epigenomics AG Corporation). Epi ProColon^®^ (also referred to as the mSEPT9 assay) became FDA-approved for CRC screening in April 2016, and it is the first blood test used with this objective. The mSEPT9 assay depends on qualitative detection by real-time PCR of methylated Septin9 that is present in abundant levels in patients with CRC. 

A prospective clinical trial in an asymptomatic screening cohort showed lower rates of sensitivity (48%) and specificity (92%) for CRC. However, this sensitivity dropped to 35% for stage I CRC and 11% for advanced adenomas, nearly eliminating its preventive role [93]. 

A next-generation test (Epi ProColon 2.0; Epigenomics AG Corporation) was recently evaluated, with a sensitivity of 48.2% to 95.6% for early-stage CRC with a variation of specificity between 80–96.7%. The blood-based mSEPT9 assay detected about half of preclinical CRC with a specificity comparable to guaiac-based FOBT in a prospective masked study. Furthermore, sensitivity of mSEPT9 for advanced adenomas was very low, thereby reducing its clinical utility [94].

Despite the diagnostic value of detecting advanced-stage CRCs (III–IV), the capacity for detecting early-stage adenomas or polyps showed low sensitivity [95].

Finally, although it was reported that mSEPT9 could be deployed for predicting CRC recurrence, metastasis, and survival, more studies are required to validate these conclusions [96,97].

New and improved epigenetic biomarkers for early detection of CRC are needed, ideally in a pre-histological stage, with a very high accuracy while using blood or stool as the substrate.

## 6. Novel Biomarkers in CRC—An Epigenetic-Based Approach

Multiple studies have highlighted the importance of epigenetic alterations in CRC, showing that they occur early and manifest more frequently than genetic alterations [17].

Ongoing efforts are trying to validate the three classes of epigenetic biomarkers for CRC screening without iatrogenicity and good compliance from the average-risk population: DNA methylation, focusing on promoters of well know genes involved in CRC pathways; Non-coding RNAs (miRNAs and lncRNAs) relevant for CRC screening; and Histone modifications of CRC [98].

### 6.1. DNA Methylation—Stool-Based Biomarkers

SFRP2 was one of the genes identified as a possible epigenetic biomarker for CRC, during studies focused on the methylation status of genes found in colonic cells present in patient feces. SFRP2 has been shown to have a sensitivity for CRC screening of 79% (95% CI: 75–82%), accompanied by an impressive specificity of 93% (95% CI: 90–96%).

Methylated SFRP2 can also spot patients with precancerous colonic polyps, with a sensitivity of 43% (95%CI: 38–49%) and a specificity of 94% (95%CI: 91–97%) [99].

The methylation status of other genes, including BMP3, SDC2, PHACTR3, SPG20, TFPI2, TMEFF2, KRAS, HPP1, SFRP2, MGMT, and WIF1, have also been investigated in fecal DNA for early detection of CRC [17,96,97,98,100].

### 6.2. DNA Methylation—Blood-Based Biomarkers

It was in the 1970s that the investigation of DNA-based biomarkers in peripheral blood began. It was found that patients with various types of cancer had tumor DNA in the serum [101].

In CRC, multiple blood-based diagnostic methylation biomarkers have been studied, including ALX4, APC, HLTF, MGMT, NEUROG1, RASSF2A, Wif-1, FBN2, PI6, TMEFF1, SDC2, TAC1, THBD, and TFPI2 [96,102,103,104]. It is likely, and certainly conceivable, that a biomarker panel of methylated genes will be translated into a clinical setting for CRC screening in the near future [105].

### 6.3. Micro RNAs—Stool-Based Biomarkers

The use of miRNA-based epigenetic biomarkers found in the stool of patients with CRC is recent. It was in 2010 that one of the first studies demonstrated the possibility of detecting miRNA in feces through a process called direct miRNA analysis (DMA). This study, in addition to demonstrating the feasibility of isolating and quantifying miRNA from feces, also revealed a dysregulation in the expression of miR-21 and miR-106a that became biomarkers for CRC screening [106]. 

In a meta-analysis performed in 2019, it was shown that pooled fecal-based miRNAs have a relatively high detection accuracy for CRC. This included 10 studies on miRNA in colon adenoma and 46 in CRC in a total of 12,827 stool-based miRNA tests. This study concluded that using different panels of miRNAs (miR-21 or miR-92a) as biomarkers results in higher CRC and adenoma detection. Moreover, the combination of these panels with FOBT or FIT may increase the specificity [107].

### 6.4. Micro RNAs—Blood-Based Biomarkers

miRNAs are protected from the action of RNases due to their size and hairpin-loop structure, and for this reason are very stable, being easily obtained from a large amount of substrates, such as blood, feces, tissues, urine, and other biological products, including tissues preserved in formalin and paraffin. Moreover, miRNAs are actively secreted by cancer cells into the blood and the digestive tract [101,103,108,109]. This property makes them attractive cancer biomarker candidates.

The detection of dysregulated miRNAs circulating in peripheral blood has been the subject of several studies in recent years [104,105,106]. Ng and colleagues led a comprehensive study where they assessed changes in miRNA expression in patients with CRC, compared to healthy individuals, in blood and tissue. In this study, it was demonstrated that miR-92a and miR17-3p detect patients with CRC with a sensitivity of 64% and 89% for each of these miRNAs, respectively, and a specificity of 70% for each of these miRNAs [110].

A study evolving 139 patients diagnosed for CRC and 132 non-CRC patient’s controls showed that expression levels in the plasma of miR-103a-3p, miR-117-3p, miR-151a-5p, miR-17-5p, miR-181a-5p, miR-18a-5p, and miR-18b-5p were meaningfully up-regulated in the plasma of CRC patients compared with healthy ones. These miRNAs can be biomarkers for blood screening of CRC patients [111].

A study carried out in plasma shows that miR-409-3p, miR-7, and miR-93 are good candidates for CRC screening because, when used together as a panel, they showed 82% sensitivity and 89% specificity in identifying CRC patients versus healthy individuals [107].

MiR-21 is another well-known oncogenic miRNA, which is considered a potential non-invasive biomarker for the early detection of CRC since it is highly expressed and its dysregulation often occurs in early stages of adenoma to carcinoma evolution [112,113]. 

In a meta-analysis study that involved diagnostic and prognostic studies based on blood-derived miRNAs, it was observed that there are several miRNA panels that could be good biomarkers for screening for CRC. The detection of miR-21 in blood can be a high diagnostic value biomarker, while the same miRNA detected in tumor tissues can be a good prognostic marker. It is becoming increasingly clear that a judicious miRNA panel can be an asset in the early detection and combat of CRC [114].

It seems improbable that a single miRNA will adequately capture the causal disease heterogeneity in colorectal polyps and cancers [115]. Therefore, numerous studies have proposed aggregating several miRNAs into a biomarker panel to improve the accuracy of CRC screening [112,116,117,118].

However, the lack of consistency between different biomarker panels from independent studies suggests that more research needs to be done in order to find the optimal grouping of diagnostic CRC miRNAs.

### 6.5. Histone Modifications

Histones regulate gene expression through changes to the three-dimensional structure of the chromatin [119].

Several studies show that histone modifications play an important role in carcinogenesis in CRC. Acetylation and methylation of H3 lysine 56, 9, and 27 are possible future epigenetic biomarkers in this and other cancers. These studies also showed that reduced values of H3K9me3 and H4K20me3 in patients’ plasma may be related to the presence of CRC [120,121]. 

In Figure 2 we present the sensitivity, specificity, strengths, and weaknesses of the available tests to screen for CRC and the tests in clinical trials that are not yet accessible (blue box).

## 7. Discussion and Conclusions

CRC is a disease with a multifactorial etiology that can be divided into sporadic or genetic forms, where three major molecular pathways are at play (CIN, MSI, and CIMP).

The development of sporadic CRC can take up to twenty years, and survival is directly associated with an early diagnosis of adenomas, polyps, or even pre-histological genetic/epigenetic aberrations.

Colonoscopy is a highly effective method for CRC diagnosis, but it is expensive and has low compliance from the average-risk population due to iatrogenic complications, particularly the relatively high risk of bowel perforation. Therefore, CT colonography or Colon Capsule Endoscopy are the alternate methods of choice.

Three decades ago, the screening of occult blood in the stool heralded a revolution in the diagnosis of CRC, as it led to earlier detection and decreased mortality.

This technique evolved and improved in accuracy, however, there are still some limitations related to this test. Namely:

(1) It only detects CRC that developed with the bleeding lesions. However, bleeding can be sporadic, and many CRC lesions might progress without bleeding;
(2) It detects lesions in a histological cancer phase, sometimes too late for an effective treatment;
(3) It requires bowel preparation;
(4) It presents many false positives due to peptic ulcers or bowel hemangiomas;
(5) It presents many false positives due to the wide use of antiplatelet, anticoagulant, or non-steroid anti-inflammatory drugs;
(6) In specific populations, it has low compliance due to religious or social reasons.

Therefore, significant efforts have been undertaken to improve CRC screening, including the development of epigenetic-based biomarkers that attempt to detect early stages of CRC. Epi ProColon 2.0 is one such example used with blood samples as the input for detecting aberrant methylation in gene promotors.

Epigenetic biomarkers comprise a recent but important body of CRC research, since epigenetic events might occur in the early stages of the oncogenic process via dysregulation of tumor suppressors or oncogenes, even before genetic mutations are observed. These may drive the sporadic transformation of colonic mucosa prior to histological manifestations induced by insults found in diets, therapeutic drugs, microbiomes, and bowel inflammatory diseases. (Figure 1).

There are many promising biomarkers covering the range of epigenetic modifications, such as miRNAs (miR-21, miR-92a, miR-17-3p or miR-96a, and others), methylation at gene promoters, including SFRP2, SFRP5, PGR, CALCA, IGFBP2, ALX4109, APC95, CDKN2A93, HLTF110, HPP1111, hMLH1110, MGMT95, NEUROG1112, NGFR, RASSF2A95, SFRP2, VIM113, and WIF195, and histone modifications (acetylation of H3 lysine 56 and di- or tri-methylation of H3 lysine 9 and 27) in CRC.

Due to the notable molecular heterogeneity observed in CRC, it will not be simple to find a single biomarker sufficient for all CRC manifestations. Therefore, a set of biomarkers applied sequentially and rationally in feces and blood appear to be the optimal strategy to effectively screen for CRC.

This review of the screening and diagnostic techniques puts in evidence that it should be possible to increase the identification of individuals with pre-cancer and cancer lesions using a rational approach to the various methods. The rational cost/benefit of the different methods needs to be considered in the equation, but it is always better to spend in screening instead of treating advanced CCR. Therefore, we envision that the collection of blood and stool from individuals can be made at the same time of FIT testing to detect CCR hemorrhagic lesions.

## 8. Future Directions

The answer to the question of what combination of CRC screening tests to choose will depend on the systematic analyses of the efficacy scores of the different tests currently available and on clinical trials. It needs to integrate information on how well they predict the risk of having CRC, and whether they are done in a sequential or random order. We foresee that the future of CRC screening will be through the application of a battery of tests adapted to the characteristics of the patient, each predicated on the different etiologies and histological diversities inherent to this tumor type. In so doing, the greatest challenge in the field can be leveraged as a clinical asset.
